# The Rapid Implementation of Ad Hoc Tele-Critical Care Respiratory Therapy (eRT) Service in the Wake of the COVID-19 Surge

**DOI:** 10.3390/jcm11030718

**Published:** 2022-01-29

**Authors:** Margarete Pierce, Steven W. Gudowski, Karsten J. Roberts, Anthony Jackominic, Karen K. Zumstein, Amanda Shuttleworth, Joshua Ho, Phillip Susser, Alomi Parikh, John M. Chandler, Ann Marie Huffenberger, Michael J. Scott, C. William Hanson, Krzysztof Laudanski

**Affiliations:** 1 Respiratory Care, Hospital of the University of Pennsylvania, Philadelphia, PA 19104, USA; margarete.pierce@pennmedicine.upenn.edu (M.P.); steven.gudowski@pennmedicine.upenn.edu (S.W.G.); karsten.j.roberts@gmail.com (K.J.R.); anthony.jackominic@pennmedicine.upenn.edu (A.J.); karen.zumstein@pennmedicine.upenn.edu (K.K.Z.); amanda.shuttleworth@pennmedicine.upenn.edu (A.S.); 2 Perelman School of Medicine, University of Pennsylvania, Philadelphia, PA 19104, USA; joshua.ho2@pennmedicine.upenn.edu (J.H.); philip.susser@pennmedicine.upenn.edu (P.S.); alomiparikh@gmail.com (A.P.); 3 Department of Neurology, Hospital of the University of Pennsylvania, Philadelphia, PA 19104, USA; john.chandler@pennmedicine.upenn.edu; 4 Penn Medicine Center for Connected Care, Hospital of the University of Pennsylvania, Philadelphia, PA 19104, USA; ann.huffenberger@pennmedicine.upenn.edu (A.M.H.); michael.scott@pennmedicine.upenn.edu (M.J.S.); hansonb@uphs.upenn.edu (C.W.H.III); 5 Department of Anesthesiology and Critical Care, Hospital of the University of Pennsylvania, Philadelphia, PA 19104, USA; 6 Leonard Davis Institute for Healthcare Economics, University of Pennsylvania, Colonial Penn Center, 3641 Locust Walk #210, Philadelphia, PA 19104, USA

**Keywords:** PENN E-LERT^®^, respiratory therapist, telemedicine, intensive care, teleICU, COVID-19, ARDS, compliance, pandemic, critical care, virtual medicine

## Abstract

A 24/7 telemedicine respiratory therapist (eRT) service was set up as part of the established University of Pennsylvania teleICU (PENN E-LERT^®^) service during the COVID-19 pandemic, serving five hospitals and 320 critical care beds to deliver effective remote care in lieu of a unit-based RT. The eRT interventions were components of an evidence-based care bundle and included ventilator liberation protocols, low tidal volume protocols, tube patency, and an extubation checklist. In addition, the proactive rounding of patients, including ventilator checks, was included. A standardized data collection sheet was used to facilitate the review of medical records, direct audio–visual inspection, or direct interactions with staff. In May 2020, a total of 1548 interventions took place, 93.86% of which were coded as “routine” based on established workflows, 4.71% as “urgent”, 0.26% “emergent”, and 1.17% were missing descriptors. Based on the number of coded interventions, we tracked the number of COVID-19 patients in the system. The average intervention took 6.1 ± 3.79 min. In 16% of all the interactions, no communication with the bedside team took place. The eRT connected with the in-house respiratory therapist (RT) in 66.6% of all the interventions, followed by house staff (9.8%), advanced practice providers (APP; 2.8%), and RN (2.6%). Most of the interaction took place over the telephone (88%), secure text message (16%), or audio-video telemedicine ICU platform (1.7%). A total of 5115 minutes were spent on tasks that a bedside clinician would have otherwise executed, reducing their exposure to COVID-19. The eRT service was instrumental in several emergent and urgent critical interventions. This study shows that an eRT service can support the bedside RT providers, effectively monitor best practice bundles, and carry out patient–ventilator assessments. It was effective in certain emergent situations and reduced the exposure of RTs to COVID-19. We plan to continue the service as part of an integrated RT service and hope to provide a framework for developing similar services in other facilities.

## 1. Introduction

Respiratory distress and acute hypoxemic failure are the primary symptoms of COVID-19 pneumonia [1]. Patients often require frequent escalation and de-escalation of respiratory therapy as the disease trajectory is highly unpredictable and labor-intensive. Providers are at a high risk of exposure due to the high labor burden of COVID-19 patient care in combination with the aerosol-based spread of the illness [2,3,4,5]. The viral dose, virulence, and individual susceptibility determine the natural history of the disease [4]. The ideal way to limit exposure is by using appropriate personal protective equipment (PPE), but institutions have often faced shortages [4,6]. Furthermore, the pandemic nature of the growth of COVID-19 has further stressed the system by creating a bottleneck in the PPE supply and restricting available medical professionals due to SARS-CoV-2 exposure [7]. Respiratory therapists (RTs) are an integral part of the U.S. healthcare system that increases the capacity of the primary intensive care unit (ICU) teams [8]. COVID-19 presents unique challenges to this service [1,7,9]. Even under optimal conditions, COVID-19 generates time-consuming tasks due to the complexity of respiratory demise [1,10]. High demand for RT skills, coupled with frequent contact with patients in settings with suboptimal PPE, can lead to increased RT occupational exposure [4]. The latter increases the number of infected individuals and further stresses the system by removing much-needed experts from the care team. Moreover, the frequent movement of RTs in hospitals operating in a pandemic may lead to patient cross-infection via the RT [11].

Telemedicine experienced explosive growth during the COVID-19 pandemic [12,13]. Some of the key drivers have been flexible delivery of care, scalability, and protection of healthcare workers in one technologically mature solution [14]. While there is research on telemedicine extending the availability of critical care specialty physicians, there are limited data on how RTs using this technology could impact patient care [15]. Despite several studies and statements indicating that telemedicine is the most flexible tool to match demand and supply, no in-depth analysis has been performed on the real-time deployment of tele-ICU services. Here, we describe a deployment of tele-ICU respiratory care services (eRT) in the wake of the COVID-19 pandemic. Our initial report covered some of the aspect of very early implementation but at that time we were not able to produce more in-depth analysis of the implementation [16]. In contrast, here we analyzed the expediency, operational model, and outcomes of eRT deployment on healthcare delivery during the pandemic in a large US healthcare system in much more depth and details. 

## 2. Materials and Methods

### 2.1. The Emergence of eRT Services

During the initial stages of the COVID-19 pandemic, we hypothesized that effective respiratory services can be delivered via telemedicine and result in the improvement of service delivery, as well as a reduction in PPE usage. Respiratory services providers in U.S. healthcare are charged with delivering the broadly understood care supporting the respiratory system in patients. They must be a graduate from a college or university with a degree in respiratory therapy and be required to pass a national board certifying examination. Their actual scope of practice is determined by local and state rules and defined by their director.

In our case, seven respiratory therapists with board certification were enlisted [9]. Four of the seven RTs (57%) hold the Adult Critical Care Specialty (ACCS) credential from the National Board for Respiratory Care (NBRC). Their average years of practice were 17 ± 6.5 years with 10 ± 6.6 years in the current healthcare system. They had no prior experience working within teleICU services.

**Figure 1:**
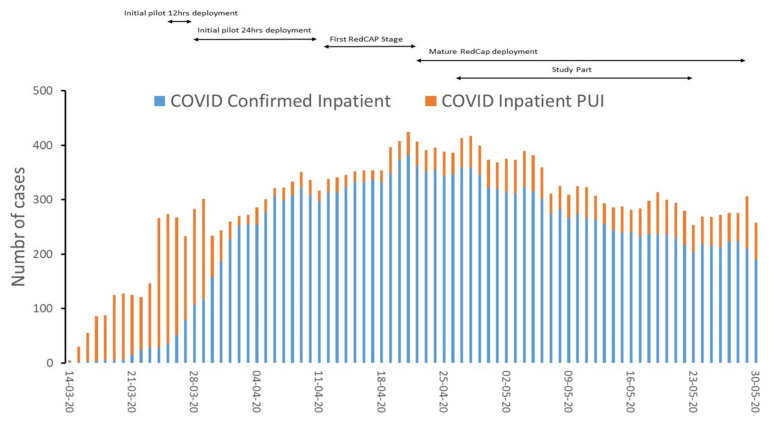
An overview of the study’s timeline, including developments in the deployment of REDCap and data collection.

The service eRT template was created at the start of the pandemic when it was predicted that ICU capacity was unable to match the expected number of COVID-19 ICU patients. The eRT pilot started on 26 March 2020, with coverage from Monday through Friday, 7 a.m.–3:30 p.m. Upon approval by leadership, physician-led training introduced five experienced RTs to the remote ICU environment within 72 h. On 31 March 2020, the eRT service was moved to a centralized location housed within the PENN E-LERT program to integrate this service with the pre-existing infrastructure of the teleICU program. The eRT was in the same room as four 24/7 teleICU nurses, a 24/7 Intensivist, day-time critical care hospitalist, and day-time anesthesiology resident. The initial intervention template included evidence-based care bundle components, such as ventilator liberation protocols, low tidal volume protocols, the surveillance of endotracheal tube occlusion, and reintubations. Initially, RTs provided feedback using a free-text template. This was followed by developing a standardized research electronic data capture (RedCAP) tool hosted at Penn Medicine to capture the eRT interventions. Coverage was expanded to 24-h coverage Monday through Friday on 6 April 2020. The eRT staff were re-allocated from in-house RT and increased to seven RTs who rotated to provide 24/7 coverage equivalent to 4.2 FTE. On 28 April 2020, the final version of the REDCap database was released and used to capture the data presented here covering May 2020 (Figure 1).

We decided to collect the data over one month as a convenience sample. Based on the pilot program observations, we aimed to collect a minimum of one thousand interventions.

### 2.2. Workflow

Within the context of the teleICU, seven respiratory therapists provided 24-h a day, 7-day a week coverage in the adult intensive care units during the COVID-19 pandemic across 5 hospitals. The audio-video interventions were completed using a teleICU workstation located remotely at PENN E-LERT. Their service was set up parallel to the ICU-based RTs covering 16 different ICUs with a nominal bed capacity of 320, resulting in an average of one RT to every twelve patients. ICUs were specialized across COVID-19 (54%) or non-COVID-19 (46%) units. The bedside team on site oversaw patients in a well-established manner, while eRTs provided a consultative service [1,8,9]. The eRTs worked within the framework of the pre-existing teleICU service at PENN E-LERT.

**Figure 2:**
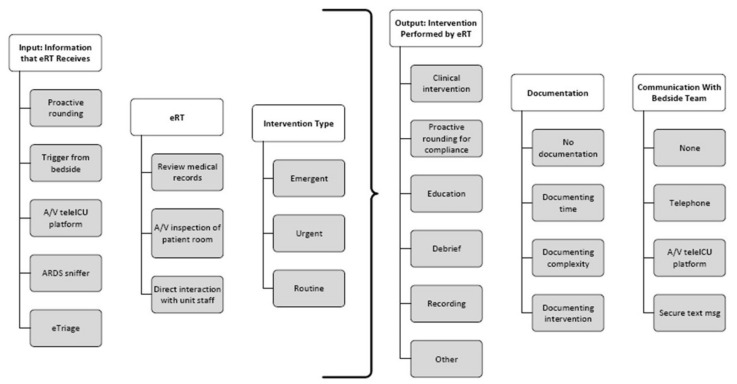
A visualization of the eRT workflow.

The eRT workflow is visualized in Figure 2. The eRT used a standardized operational procedure sheet to detect issues during medical record review, direct audiovisual patient inspection, or direct interaction with in-house staff, driven by eRT (proactive rounding). Requests for specialized RT services could come from the ICUs (site-trigger). Requests for eRT services could also be automatically generated using an alert from -the medical record or an electronic “sniffer” aimed at detecting ARDS or sepsis [17,18,19]. Finally, other teleICU staff could request the attention of the eRT to certain problems they encountered while performing their tasks (eTriage). Other request methods included an in-room emergency button that would directly contact PENN E-LERT. These represent a small fraction of all events.

Review of electronic medical records, a direct remote audio–visual inspection of the patient room and ventilators, and direct interaction with unit staff could be concurrently employed to accomplish a task. If the need for in-house eRT intervention was determined, the eRT reached out to their ICU-based counterpart in the unit with a suggestion.

The expediency of response was deemed routine if the required response time was within 2 h, urgent for fewer than 30 min, or emergent for immediate response.

The response included several standardized tasks with different scopes. One option, eRT defer, was when the primary team deferred ventilator checks to be completed by the eRT service rather than by the in-person team. Ventilatory support refers to respiratory deterioration in mechanically ventilated patients. Respiratory decline interventions were performed for non-intubated patients due to respiratory demise. Compliance was a task aimed at synchronizing clinical activity with medical documentation. Extubation risk pertained to a standardized protocol created for all patients deemed high-risk for extubation, SAT/SBT surveillance of patients for a spontaneous breathing trial or extubation, or surveillance of newly extubated patients [20,21]. ARDS referred to the implementation of an ARDS/low tidal volume bundle [20,21,22]. All eRTs could note any interventions not encapsulated above using free text.

The eRT determined the need to communicate with the bedside team. If communication was deemed necessary, telephone, secure text message, or audio-video communication via the teleICU platform were employed. The eRT recorded the time and complexity of the task.

### 2.3. Data Collection

The data were collected between 1 May 2020 and 31 May 2020 using the final electronic REDCap database created from an earlier version of the REDCap survey (Supplementary File S1) [23,24]. Prototype databases used handwritten notes initially created by eRTs to capture events during the shifts between 26 March 2020 through 27 April 2020. Data were tabulated at the end of the period. Missing data were left as is and omitted from analysis.

The study’s primary outcome was the number of interventions taken by teleICU eRT service in lieu of bedside RT service. The secondary outcome was the amount of PPE saved due to substituting traditional RT service with eRT.

### 2.4. Statistical Analysis

Descriptive statistics were primarily used. The Shapiro–Wilk W test and distribution plots were used to test the normality of distribution variables. Homogeneity of variance was evaluated with Levene’s test. Parametric variables were expressed as mean ± SD and compared using Student’s *t*-test. For non-parametric variables, median (Me) and interquartile ranges (IRs) were shown with Mann–Whitney U statistic employed to compare such variables. The data were analyzed as dependent, paired samples. A double-sided *p*-value less than 0.05 was considered statistically significant for all tests. Statistical analyses were performed with the Statistica 11.0 (StatSoft Inc., Tulsa, OK, USA).

## 3. Results

### 3.1. eRT Service Healthcare Delivery Model

**Figure 3:**
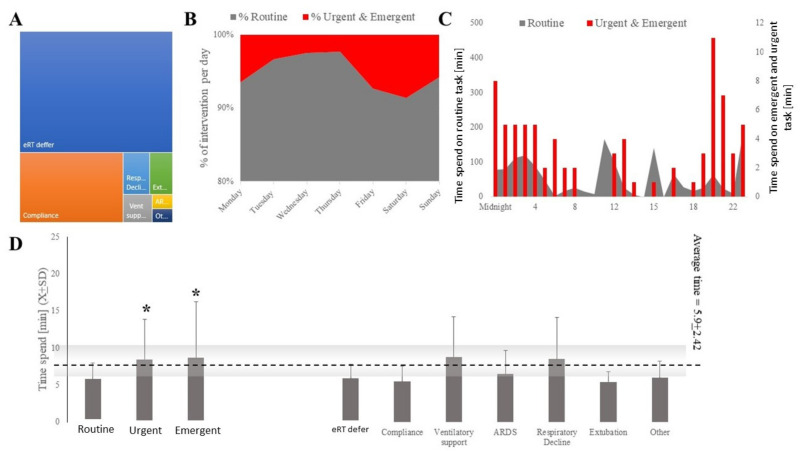
eRT interventions were mostly categorized as activities deferred from bedside or best practice compliance (**A**) and were more likely to occur during the weekend or at night (**B**,**C**). Less time was spent by eRT on routine tasks compared to those categorized as urgent or emergent (**C**,**D**). * denotes statistical significance at the level of 0.05.

A total of 1548 interventions took place between 1 May 2020 and 31 May 2020, and 93.86% of the interventions were routinely related to the established workflows, 4.71% were urgent, and 0.26% were emergent. The majority of the interventions were related to RT deferring activities to eRT such as ventilator checks or compliance with best practice protocols (Figure 3A). Higher demand for eRT services occurred during weekends and nighttime hours (Figure 3B,C). The number of interventions somewhat trailed the demand from COVID-19 in the system. 

The bulk of the interventions were triggered during proactive rounding (59.7%), site-triggered (23.4%), or by the teleICU staff (eTriage; 4.9%), directing the attention of an eRT to clinical problems (Table 1). There were statistically significant differences (H(2;1523) = 39.36; *p* < 0.00001) in times devoted to the tasks depending on the task’s expediency. The time spent on routine tasks (t_routine_ = 5.8 ± 2.15) was the shortest, while urgent (t_urgent_ = 8.5 ± 5.38) and emergent (t_emergent_ = 8.8 ± 7.5) tasks took about the same amount of time but longer than routine tasks (Figure 3C). The average intervention took 5.97 ± 2.49 min, but it varied statistically by the different natures of each task (H(6;1525) = 89.89; *p* < 0.0001; Figure 3C,D). There were significant differences between the amounts of time spent by different eRTs on the tasks (H(6;1530) = 491.35; *p* < 0.000).

**Figure 4:**
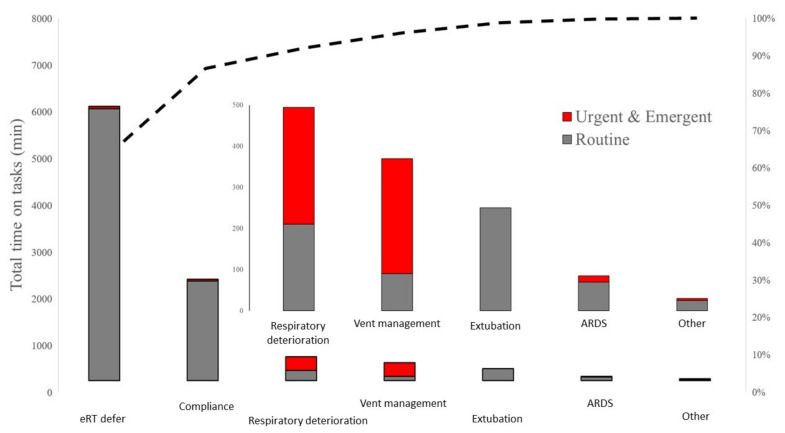
eRT spent the most time on activities that were deferred from RT, routine ventilator assessment and assuring compliance with best practice standards.

In 16% of all the interventions, no communication with the bedside took place. The communication between eRTs and ICU-based RTs took place in 66.6% of all the interventions, followed by communication with house staff (9.8%), advanced practice providers (APP; 2.8%), and nurses (2.6%). Most communication involved a single provider at the bedside, but, in 2.6%, several stakeholders were involved. Most of the interactions took place over the telephone (88%), followed by secure text message (16%), audio-video communication via the teleICU module platform (1.7%), and the remaining were negligible (The eRT’s recommendation was followed in 92% of the cases. In 4% of the cases, they were only received, with the remaining 4% being missing data. The majority of the interventions involved deferring a routine ventilator assessment or assuring compliance of orders with ongoing ventilator settings (Figure 4). The clinical tasks focused on worsening respiratory status, extubation screens, and advanced ventilator management. Of note, ventilatory management or respiratory deterioration were deemed urgent or emergent in over 50% of the cases (Figure 4).

### 3.2. The Involvement of eRT Services with COVID-19 and Non-COVID-19 Patients

**Figure 5:**
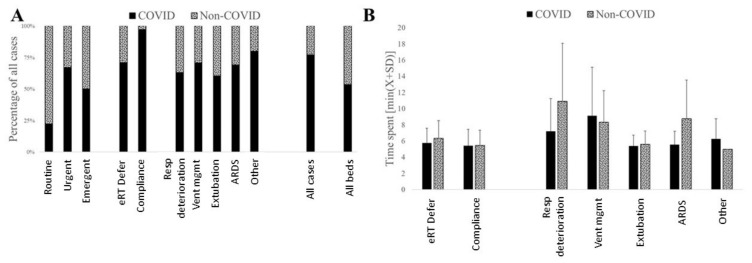
The majority of all eRT interventions were related to COVID-19 patients (**A**). Similarly, more time was spent by eRT when providing care to COVID-19 patients when compared to their non-COVID-19 counterparts. (**B**). Averting respiratory deterioration and supporting ventilator management in ARDS cases was the largest time spent in non-COVID-19 patients.

Seventy-four percent of the interventions were related to COVID (Figure 5A). The frequency of interventions was as expected, with a 54% vs. 46% ICU-bed type split between the COVID and non-COVID patients observed in this healthcare system. The average COVID-19-related burden was higher for all the eRT services in terms of time spent providing care to COVID-19 vs. non-COVID-19 patients (t_COVID-19_ = 5.8 ± 2.25 vs. t_COVID-19_ = 6.0 ± 3.05 U(166426) = −4.21; *p* < 0.0001). The time spent on different tasks was not different between the COVID and non-COVID-19 patients (Figure 5B).

## 4. Discussion

Here, we demonstrated that the deployment of eRT could be achieved rapidly and be clinically beneficial and staff-preserving. Furthermore, this innovative process leverages teleICU and RT solutions, creating new value that was not practically demonstrated at this level before.

Traditionally, RTs provide care based on physician orders and pre-existing protocols within the standardized institutional protocols [8]. Interactions and rounding with physicians are crucial aspects of the RT role, in addition to the daily evaluations of the appropriateness of ordered therapies [25]. The tasks completed by eRTs included, but were not limited to, ventilator checks, virtual assessments of patients experiencing respiratory compromise either while intubated or non-intubated (e.g., non-invasive positive pressure ventilation or humidified high flow nasal cannula), compliance-based medical documentation, extubation screenings, SAT/SBT, and surveillance of newly extubated patients. The eRT is limited by the inability to carry out tasks in person. However, the eRTs could instruct nursing and physician staff in the room to complete a task with virtual supervision and provide education remotely when RTs at the bedside were unavailable. The eRTs also provided surveillance during emergent situations, such as intubation and CPR.

Over 1500 interventions resulted in avoiding several near-miss events and improved patient care, as demonstrated by the ability of an eRT to intervene early, often before the bedside RT. Of note, the eRTs achieved this by utilizing proactive electronic rounds and by responding to clinical cues from the bedside staff, the teleICU system, or the teleICU staff. No communication problems during the studied period were described despite prior reports of such [26,27]. In addition, they intervened upon non-compliance with a low stretch recommendation for the ARDS before our electronic system triggered an alarm. We could not quantify this intervention’s effect, but, undoubtedly, there is a clear patient benefit since delaying the introduction of the low stretch protocol in ARDS for 24 h doubles the risk for mortality [20]. A review of the free-text comments revealed eRTs detected vent disconnection events, decreased patency of the endotracheal tubes, and advised bedside providers on the overall handling of respiratory emergencies. The ability of eRTs to manage emergent and urgent problems is particularly important since this was the initial impetus for the program’s creation. Not only did eRTs meaningfully increase patient safety but they also demonstrated flexibility in being self-deployed or called upon to a different part of the hospital equipped with remote teleICU, which is not achievable by traditional in-house staffing models. The eRTs were also frequently employed during high-risk pre-extubation procedures, serving as safety officers or monitoring patients post-extubation. Considering that COVID-19 pneumonia presents incredibly complicated extubation risks due to the increased tenacious secretions and variable trajectory of respiratory demise, their participation improved healthcare delivery significantly [1,2,5,20,28]. Finally, eRTs helped with implementing novel techniques, such as the helmet CPAP.

Employment of eRTs reduced the exposure of medical professionals to COVID-19 patients or trans-patient COVID-19 transmission via medical staff. The People’s Republic of China data suggested a 0.72% risk of COVID-19 acquisition amongst medical personnel [2,3,11]. Replacing the 511 documented instances (RT deferred activities, advanced ventilator management, worsening respiratory status, extubation check, and surveillance of the newly extubated) when an eRT instead of an RT interacted with patients, 3.6% of COVID cases could be avoided in our model assuming a 0.72% infection rate. This estimate is based on the short exposure to the COVID-19 environment. However, several interventions performed by eRTs were rather long. Therefore, the minimization of COVID-19 exposure is probably underestimated. Furthermore, teleICU will not eliminate the risk of transmission considering specific procedures have to be done manually [4,5,6]. Furthermore, utilizing eRTs reduces the utilization of PPE. This financial effect is not large, yet preserving PPE has a tremendous effect on staff morale and overall hospital function [28].

The eRT’s role is very flexible if the remote system is deployed in the physical ICU. Alternatively, the reach of the teleICU can be expanded using a mobile audio–visual unit [27]. The novel development of eRT services was triggered by an attempt to match the expected demand for respiratory services due to the pandemic-triggered demand [14]. The provider and eRT frequently reported flexibility because the eRT can switch from one location to another while troubleshooting a problem. This system minimizes the time needed for the physical movement of bedside therapists while preserving other practitioners for other tasks [29]. The indirect effect of eRTs on the healthcare system capacity resulted in a modest reduction in the RT-to-patient ratios within units and lessened the effect of a COVID-19-related therapist shortage. If the teleICU infrastructure exists, all these effects could be quantified through significant financial benefits for healthcare systems without significant additional investment. Moreover, as the availability and affordability of the teleICU platform continue to progress, our model represents a universal principle of augmenting hospital services, especially during seasonal increases in demand or emergencies [1,14,30].

Finally, improved compliance with evidence-based protocols improves mortality [31]. The eRT’s role in ensuring compliance was particularly high in COVID-19 patients in our study. In addition, the rapid deployment and adjustment of the clinical protocol in the wake of a pandemic could likely lead to non-adherence. Finally, engaging the bedside staff with compliance-related issues had an educational value. Critical care certified eRTs can serve as mentors, coaching a novice through difficult situations, troubleshooting complex equipment, and supporting critical thinking skills.

Our quality and assurance project was done in a healthcare system with a well-established teleICU presence. Other less established systems may face culture and deployment issues. The development of eRTs may encounter limitations since not all tasks may be performed effectively in all systems. Developing such a system during a pandemic helps us overcome several barriers, including administrative inertia, implementation barriers, and leadership buy-in. The cost of the program deployment was low for us since we already had an existing infrastructure, but this may not be the case for other programs. Finally, measuring impact is a challenging task. Here, we utilized surveys to determine the impact of the eRT service. However, connecting specific interventions to patient outcomes or performing limited field studies could better define the effect of eRT on mortality and morbidity. Some of these outcomes and performance measurements may be specific to a facility. Additional research is needed to define the role of eRT further and measure its effectiveness when not under the stress of a pandemic. Our hospital never reached saturation conditions, so it is unclear how it would perform under such conditions [30].

## 5. Conclusions

Here, we described the successful augmentation of the existing hospital RT services with eRT services in a location with an established teleICU program (PENN E-LERT) during the COVID-19 pandemic. We demonstrated several benefits, such as improved protocol-driven patient care, elevated healthcare system performance, and reductions in expenses. Resultantly, we aim to integrate the eRT service into future practice.

## Figures and Tables

**Table 1:** Frequency of different sources for eRT interventions.

Intervention	Total Count	% of Total	% Cumulative
Proactive rounding	1024	59.67	59.67
Site-trigger	405	23.6	83.9
eTriage	84	4.89	88.81
Sniffer/Dashboard Tool (ARDS, sepsis, etc.)	11	0.6%	88.81
Push Button	3	0.17%	88.81
Virtual consult	1	0.1	89.04
Other (place a note in Clinical Comment)	1	0.1	89.10
Missing	187	10.89	100.0

## Data Availability

The datasets used and/or analyzed during the current study are available from the corresponding authors on reasonable request.

## Supplementary Materials

The following supporting information can be downloaded at: https://www.mdpi.com/article/10.3390/jcm11030718/s1, Supplementary File S1: REDCap survey.
