# Supplementary material for: The Rapid Implementation of Ad Hoc Tele-Critical Care Respiratory Therapy (eRT) Service in the Wake of the COVID-19 Surge
Source: J Clin Med. 2022 Jan 29;11(3):718. doi: 10.3390/jcm11030718 (PMC8837076; doi:10.3390/jcm11030718)
Supplement: Supplementary file 1 [file jcm-11-00718-s001.zip › jcm-1520696-supplementary.pdf]

Penn Elert

Resize font:

Please complete the survey below after each intervention

Thank you!

Date/Time

Now D-M-Y H:M

Initials of Provider

2 characters remaining

Patient MRN
Put 0000001 for Aggregated time for hyperoxia (six ZEROS)
Put 0000002 for Aggregated time for DVT (six ZEROS)
Put 0000003 for Aggregated time for GI (six ZEROS)
Put 0000004 for Aggregated time for APACHE (six ZEROS)
Put 0000005 for Aggregated time for BPH (six ZEROS)
Put 0000006 for Aggregated time for Orders Reconciliation (six ZEROS)

Your role?

- ○ eMD
- ○ eAPP
- ○ eRN
- ◉ eRT
- ○ eTHC
- ○ eRes
- ○ eMS

reset

Expediency of intervention

- ○ Routine
- ○ Urgent
- ○ Emergent

reset

Did you respond to:

- ○ Push Button
- ○ Virtual consult
- ○ eICU staff triage
- ○ Phone call (from site)
- ○ Proactive rounding
- ○ Sniffer/Dashboard Tool (ARDS, sepsis, etc)
- ○ eCareManager Generated Alert

reset

Site originated

Communication with on-site person

- ☑ No communication (DEFAULT)
- ☐ Escalation to Penn eLert staff
- ☐ Medical Director
- ☐ Attending
- ☐ Resident
- ☐ APP/CRNA
- ☐ RN
- ☐ RT
- ☐ Medical resident/fellow
- ☐ Family
- ☐ Administrative staff
- ☐ Other (place a note in Communication)

Primary focus of the interaction

- ☐ Clinical intervention
- ☐ Proactive rounding/Q&S
- ☐ Education
- ☐ Debrief
- ☐ Recording
- ☐ Other (place a note in Clinical Comment)

Task Scope

- ☐ Worsening status / Advanced ventilator/BIPAP management
- ☐ Worsening status on non intubated/BiPAP patient
- ☐ Extubation/High risk check list.
- ☐ Newly extubated surveillance
- ☐ (PLACEHOLDER _ DO NOT CHECK)
- ☐ Deferring scheduled checks to the remote team
- ☐ Prioritize SBT/SAT
- ☐ TJC compliance with PennChart orders
- ☐ ARDS & low stretch protocols issues
- ☐ Other (place a note in Clinical Comment)

COVID related

- ○ Yes
- ○ No

reset

Recommendation placed in EPIC

- ○ Yes
- ○ No

reset

Approximate Total time spent on chart (min)

- ◉ 5
- ○ 10
- ○ 15
- ○ 20
- ○ 25
- ○ 30
- ○ 40
- ○ 50
- ○ 60
- ○ more than 1hour

reset

Complexity

Less compless | Nominal for my expertise and job decription | More Complex

Change the slider above to set a response

reset

Clinical Comment

200 characters remaining

Expand

Submit

Powered by REDCap
